# One-Step Electrochemical Dealloying of 3D Bi-Continuous Micro-Nanoporous Bismuth Electrodes and CO_2_RR Performance

**DOI:** 10.3390/nano13111767

**Published:** 2023-05-30

**Authors:** Wenqin Lai, Yating Liu, Mingming Zeng, Dongmei Han, Min Xiao, Shuanjin Wang, Shan Ren, Yuezhong Meng

**Affiliations:** 1 The Key Laboratory of Low-Carbon Chemistry & Energy Conservation of Guangdong Province, State Key Laboratory of Optoelectronic Materials and Technologies, School of Materials Science and Engineering, Sun Yat-sen University, Guangzhou 510275, China; laiwq3@mail2.sysu.edu.cn (W.L.); liuyt79@mail2.sysu.edu.cn (Y.L.); zengmm5@mail2.sysu.edu.cn (M.Z.); handongm@mail.sysu.edu.cn (D.H.); stsxm@mail.sysu.edu.cn (M.X.); wangshj@mail.sysu.edu.cn (S.W.); 2 School of Chemical Engineering and Technology, Sun Yat-sen University, Zhuhai 519000, China

**Keywords:** nanoporous, dealloying, 3D architectures, CO_2_ reduction, formate

## Abstract

The rapid development of electrochemical CO_2_ reduction offers a promising route to convert intermittent renewable energy into products of high value-added fuels or chemical feedstocks. However, low faradaic efficiency, low current density, and a narrow potential range still limit the large-scale application of CO_2_RR electrocatalysts. Herein, monolith 3D bi-continuous nanoporous bismuth (np-Bi) electrodes are fabricated via a simple one-step electrochemical dealloying strategy from Pb-Bi binary alloy. The unique bi-continuous porous structure ensures highly effective charge transfer; meanwhile, the controllable millimeter-sized geometric porous structure enables easy catalyst adjustment to expose highly suitable surface curvatures with abundant reactive sites. This results in a high selectivity of 92.6% and superior potential window (400 mV, selectivity > 88%) for the electrochemical reduction of carbon dioxide to formate. Our scalable strategy provides a feasible pathway for mass-producing high-performance and versatile CO_2_ electrocatalysts.

## 1. Introduction

The electrochemical reduction reaction of CO_2_ (CO_2_RR) is considered a promising method for producing useful carbon-containing compounds [1,2,3]. Formate or formic acid is an important chemical intermediate widely used in many industrial processes. It holds great potential as a direct fuel for automobiles and as a future hydrogen storage compound. It is currently considered one of the most economically valuable fuel products resulting from CO_2_ electrocatalytic reduction [4,5]. Bismuth (Bi) has drawn particular attention for its ability to selectively convert CO_2_ into formic acid, its low toxicity, low cost, and good stability as well as low HER affinity and its strong binding to *OCHO species compared to other metal-based catalysts (Sn, Pb, Pd et al.) [6,7,8]. However, Bi-based electrocatalysts are still needed to overcome the two-electron energy barriers for CO_2_ activation to enhance selectivity, current density and expand the range of high efficiency potential [9,10,11]. To address these issues, a variety of nano-bismuth electrocatalysts including Bi nanoparticles (spherical or other shape) [10,12], Bi single atom [13], Bi nanosheets [14,15,16], Bi nanotubes [17], and Bi nano-dendrite [18] have been rationally designed. Multitudinous nanostructure building strategies are introduced such as refining nanocrystal size, tailoring crystal facets, and surface functionalization with molecular coatings on the contact region [19,20]. However, the construction of 3D nanostructures is still under limited study. Previous works were generally concentrated on combing nano-sized bismuth with 3D supporting substrates to enlarge the active surface area [21,22,23], which increased current density but often failed to reach high faradaic efficiency and a wide potential range. Therefore, it is still a challenge to create new types of 3D bismuth nanostructures with tunable fine structure at a nanoscale level.

Dealloying is an efficient top–down method for producing free-standing 3D porous metals with a bi-continuous ligament–pore structure by selectively etching one or more active metal elements from alloys [24,25,26,27]. These monolithic bodies of nanoporous metals are widely used in the fields of actuation, sensing, and energy storage due to their large interfacial areas, tunable structure sizes, and good mechanical properties [28]. Although they possess 3D hierarchical pore structures that facilitate interfacial mass transfer and are rich in high-density under-coordinated active sites, the potential of these self-freestanding inter-connected ligament nanostructures for catalytic applications remains largely unexplored. These materials have significant potential for industrial production [29,30,31].

In this work, a production-scalable one-step electrochemical synthesis approach to fabricate a self-organized bi-continuous micro-nanoporous bismuth electrode (np-Bi) is reported. A characteristic free-standing 3D bi-continuous nanoporous pure Bi catalysis network with controllable geometric structures is created. The three-electrode H type cell electrocatalytic CO_2_RR tests of np-Bi showcase the exceptional electrochemical performance for the reduction of carbon dioxide to formate, with high faradaic efficiency (92.6%) and a superior potential window (∼400 mV, selectivity > 88%),. In addition, the np-Bi catalyst also exhibits long-term stability for up to 12 h.

## 2. Materials and Methods

### 2.1. Preparation of Np-Bi Electrode

Ingots of single-phase Pb_85_Bi_15_, Pb_88_Bi_12_, Pb_92_Bi_8_, and Pb_98_Bi_2_ parent alloy were prepared separately by mixing Pb and Bi powder (99.999% purity, Shanghai Aladdin Bio-Chem Technology Co. Ltd., Shanghai, China) with an atomic ratio under argon atmosphere in a resistor furnace at 450 °C. The ingot was vacuum-sealed into a quartz glass tube, and then melting/solidification was repeated more than three times followed by quenching in water to maintain its geometric composition uniformity. Subsequently, it was cold-rolled to an alloy foil, annealed for 24 h at 130 °C under argon atmosphere in a tube resistor furnace, and precisely cut into 5 × 5 × 0.4 mm^3^ cuboids. The electrodes were fabricated by attaching an as-prepared alloy foil to a copper plate, which was subsequently sealed in epoxy, only exposing one face of the alloy cuboids.

Electrochemical dealloying was performed in a conventional three-electrode system with a platinum (Pt) counter electrode and Hg/Hg_2_SO_4_ (MSE) reference electrode. The cell was settled in a circulating bath with a flow of heating/cooling water to enable temperature control of 10, 25 or 45 °C during the electrochemical process. Before dealloying, the Pb-Bi parent alloy foil was washed with alcohol to remove any surface contaminants. The Pb_98_B_i2_, Pb_92_Bi_8_, Pb_88_Bi_12_, and Pb_85_Bi_15_ alloys were dealloyed under a constant electrode potential of −0.55V (vs. MSE) for 1200s, resulting in the formation of np-Bi layers with a thickness of 75–80 µm. The catalyst mass of the as-dealloyed np-Bi_2_, np-Bi_8_, np-Bi_12_, and np-Bi_15_ electrodes were calculated based on Faraday’s equation to be 0.418 g, 1.645 g, 2.439 g, and 3.023 g, respectively. Subsequently, the as-dealloyed np-Bi samples were immersed in ultrapure deionized water for half an hour and washed several times prior to further electrochemical measurements

### 2.2. Electrochemical Measurements

All the electrocatalytic CO_2_RR tests were performed on a three-electrode H type cell using CHI-660e (CH Instruments, Shanghai, China) workstation. The liner sweep voltammetry (LSV) measurements were conducted in 0.1 M KHCO_3_ electrolyte saturated with N_2_ or CO_2_. The voltage ranges from −0.8 to −2.0 V (vs. SCE), and the scan rate was 50 mV s^−1^. The electrochemical surface area (ECSA) of different electrodes was calculated from the cyclic voltammetry (CV) experiments. CV tests were performed within a non-faradaic processes potential range (−1.0 V to −0.8 V vs. SCE) in N_2_ saturated 0.1 M KHCO_3_ electrolyte. The scan rates were set to 10, 25, 50, 75, 100, 125, 150, 175 and 200 mV s^−1^, respectively. The double-layer capacitance (C_dl_) of the working electrodes was estimated by plotting the capacitive current density at −0.97 V (vs. SCE) against the scan rates. The measured slope of the linear regression differences between the electrodes referred to the net C_dl_ of catalysis. The ECSA value was in direct proportion to the ratio of C_dl_. The electrochemical impedance spectroscopies (EIS) measurements were carried out in N_2_ saturated 0.1 M KHCO_3_ electrolyte with 5 mV amplitude in a frequency range from 100 kHz to 0.1 Hz.

All the applied potentials were calibrated as reversible hydrogen electrode (RHE) potentials by using the Nernst equation as below.

E(vs. RHE) = E (vs. SCE) + 0.2412V + 0.0591 × pH(1)

Electrocatalytic CO_2_RR tests were conducted in a homemade H-type electrolysis cell separated by a Nafion 117 membrane. Each compartment of the sealed cell contains 30 mL of 0.1 M KHCO_3_ electrolyte (Shanghai Aladdin Biochemical Technology Co., Ltd., Shanghai, China, 99.99%). The as-prepared np-Bi was directly used as the working electrode with Hg/HgCl_2_ (saturated KCl) electrode placed in a cathode chamber, and the Pt electrode was put in an anode chamber. The geometric surface area of the np-Bi electrodes is 0.25 cm^2^. High-purity CO_2_ gas was purged into the electrolyte in each compartment at a flow rate of 40 mL min^−1^ for more than 30 min before the electrocatalytic experiment. The pH values of the electrolyte were measured to be ~6.8 after saturation by CO_2_. Each chamber was kept stirring at 1000 r min^−1^ during electrolysis. The applied potential of the catalytic process was set to −1.5 V, −1.6 V, −1.7 V, −1.8 V and −1.9 V (vs. SCE), respectively. The catalytic time was 1 h, and the liquid phase products of the cathode cell were collected for quantitative analysis using a ^1^H NMR (Bruker Avance III, 500 MHz) after the electrocatalysis.

Typically, 500 µL of catholyte was uniformly mixed with 100 µL of deuterated water (D_2_O) and 200 µL of internal standard of anhydrous dimethyl sulfoxide (DMSO, 99.9%). A water pre-saturation method was applied to the ^1^H spectrum, and the formate calibration curve was determined by the internal standard (vs. 5 mM/L DMSO) method of using various concentrations (0.1, 0.5, 1.0, 2.0, 5.0 and 10 mM) of sodium formate (HCOONa, 99.99%). The faradaic efficiency (FE) of specific products was calculated via the following equation:

(2) $${FE}_{HCOOH}=\frac{nzF}{Q}\times 100\%$$

where F is the Faraday constant and Z indicates the number of electron transfers. The amount of charge Q is the total electricity consumed during the electrolysis process.

## 3. Results and Discussion

### 3.1. Fabrication and Characterizations of Nanoporous Bismuth (Np-Bi) Electrodes

In our previous study, it has been discovered that nanoporous Bi (np-Bi) can be converted into a Bi nanowire structure in a specific alloy composition through a designed electrochemical dealloying [32]. This suggested that modifications of the millimeter-size geometric porous structure from micrometer to nanometer scale can be achieved by simple changing the alloy composition. While the np-Bi nanowire matrix structure is too fragile to meet our goal of creating a free-standing 3D pure catalysis electrode due to its low bismuth content (obtained through dealloying from Sn_99_Bi_1_ parent alloy), Sn-Bi alloys with higher Bi content could causes the ligament size to split into two distinct scales, resulting in insufficiently small nanopores and disintegrated structures that cannot maintain the high activity required for the two-electron process of carbon dioxide reduction. Therefore, a suitable parent alloy and a designed alloy composition is essential to create a stable and active catalyst with the desired nanoporous structure.

**Figure 1:**
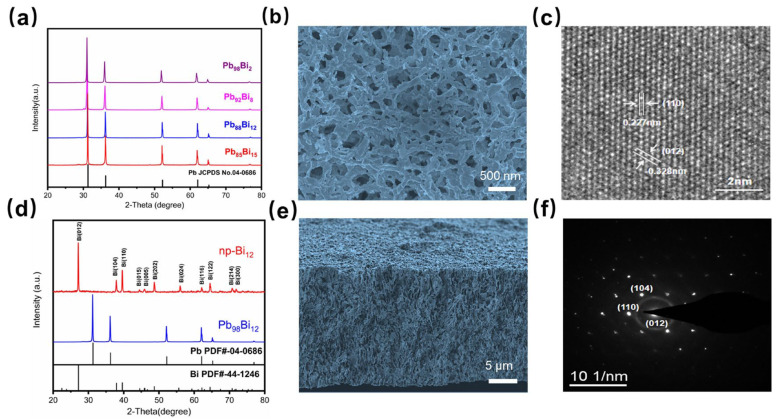
(**a**,**d**) XRD patterns of as-prepared Pb-Bi parent alloys and np-Bi_12_ electrodes before and after dealloying; (**b**,**e**) SEM images of top-view and the cross-section of as-prepared np-Bi_12_ electrodes; (**c**,**f**) high-resolution transmission electron microscopy (HRTEM) images and selected area electron diffraction (SEAD) pattern of np-Bi_12_.

In this study, four different Bi contents of Pb-Bi parent alloy were selected to investigate the effect of the component on the dealloyed nanostructure. Using the LSV curves (Figure S1, Supplementary Materials) of the parent alloy as guidance, the dissolution potential of the pure bismuth in 0.5 M HNO_3_ aqueous solution is more positive than the Pb-Bi parent alloy (>500 mV), suggesting the bismuth is more stable than the lead in an electrochemical environment. The constant dealloying potential is set at −0.55 V vs. MSE in which the current density reaches 1 mA cm^−2^. The XRD patterns of the as-prepared Pb-Bi parent alloy sample (Figure 1a) display a typical single Pb solid solution phase consistent with the phase diagram. Specifically, the three major peaks at 31.305°, 36.27°, and 52.23° are indexed to the (111), (200), and (220) patterns of cubic Pb (JCPDS, 04-0686), respectively. Other peaks are also well consistent with the JCPDS card, and no peaks of oxides of Pb or Bi are observed. The crystal phase of the fully-dealloyed np-Bi is confirmed (Figure 1d), which shows completely different patterns with three main peaks at 27.2°, 38.0°, and 39.6°, which match well with the (012), (104), and (110) faces of rhombohedral Bi (JCPDS, 44–1246). The narrow peaks exhibit the high crystalline structures and single phase of np-Bi. Energy-dispersive X-ray spectroscopy (EDX) of the as-dealloyed np-Bi (Figure S2, Supplementary Materials) shows that none of the Pb element is remained inthe porous structure, which further confirms the high purity of the synthesized np-Bi catalyst. High magnification top-view SEM images (Figure 1b) reveal an open interpenetrating ligament-pore structure of np-Bi_12_ with a uniform pore size of 280 nm and average ligament size of 290 nm.

**Figure 2:**
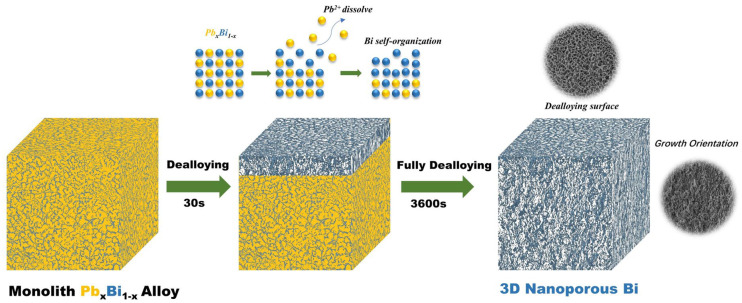
Schematic illustration of the dealloying and geometric growth of 3D nanoporous bismuth.

The HRTEM image (Figure 1c) revealed well-resolved 2D lattice fringes with d spacings of 0.227 and 0.328 nm, which coincided with the (110) and (012) lattice planes in the rhombohedral Bi structure. The SAED pattern further verifies the single-crystalline nature of np-Bi. (Figure 1f). The cross-sectional SEM images (Figure 1e) reveal a distinct growth pattern of the nanoporous structure that differs from the top-view images. The top-view images show a uniformly distributed two-dimensional diffusion-like pattern of ligament framework, while the cross-sectional images demonstrate a longitudinal, interwoven nanowire structure growing downwards from the dealloying surface (Figure S3, Supplementary Materials). This growth behavior deviates from traditional dealloying methods, which typically result in a uniform front-cross section [33,34]. Suggesting this electrochemical dealloying generates a unique morphology of the nanoporous structure with a preferred growth orientation in three dimensions (Figure 2). During the dealloying process, Pb atoms were selectively oxidized to form Pb^2+^ with a distinct hydrogen bubble occurred on the counter electrode surface. After the layer by layer dissolution of the solid–liquid interface, the remaining Bi atoms first underwent diffusion and agglomeration, which was followed by recrystallization. Finally, it stretched in three dimensions, ultimately formed a nanoporous structure. This phenomenon can be attributed to the high diffusion rate of bismuth atoms on the dealloying surface, which enables their long-range diffusion even in low bismuth content solid-solution alloys. The close atomic number proximity of lead and bismuth ensures their similar diffusivity during the dealloying and reorganization process, resulting in a closely knit structure from the np-Bi ligaments to the pore channels, and the monolith nanostructure is reserved [35].

The surface diffusion ability of stable component atoms at the dealloying interface is directly affected by the dealloying temperature, resulting in a change of nanometer size. The effects of dealloying temperature (10 °C, 45 °C, 80 °C) were investigated on Pb_98_Bi_2_ alloy (Figure S4, Supplementary Materials). At a dealloying temperature of 10 °C, a sparse hierarchical three-dimensional porous foam structure is formed with a wide pore size distribution, including some large pores of 1.2 µm, as well as numerous small pores between 300 and 600 nm. Additionally, a root-like nanowire structure appears on the ligament with a diameter of about 30 nm. When the dealloying temperature is increased to 45 °C, the resulting structure is still a hierarchical three-dimensional porous foam but shows a greater differential hierarchical structure. Macropores rapidly grow with a wide distribution of pore size of 2–10 µm, and the average macropore size increases to 5.59 µm, leading to an increase in pore size with an average size of 830 nm. Additionally, the ligament size grows to 760 nm, reflecting approximately a six-fold increase compared to the previous value of 130 nm at 10 °C. At 80 °C, the dealloying process results in a porous structure similar to nickel foam, lacking differentiation in structure. Fine nanowire branches and granular convex structures are absent, and the pore walls change smoothly. Pore sizes range from 2 to 25 µm, with no pores smaller than 1 µm observed. The maximum observable pore size is 27 µm, and the average pore size is 8.6 µm, with an average pore wall thickness of 4.8 µm. This demonstrates that the size of the nanoporous structures increased continually with the rising dealloying temperature, enabling the control of the nanoporous structure size across a wide range from nanometers to micrometers.

**Figure 3:**
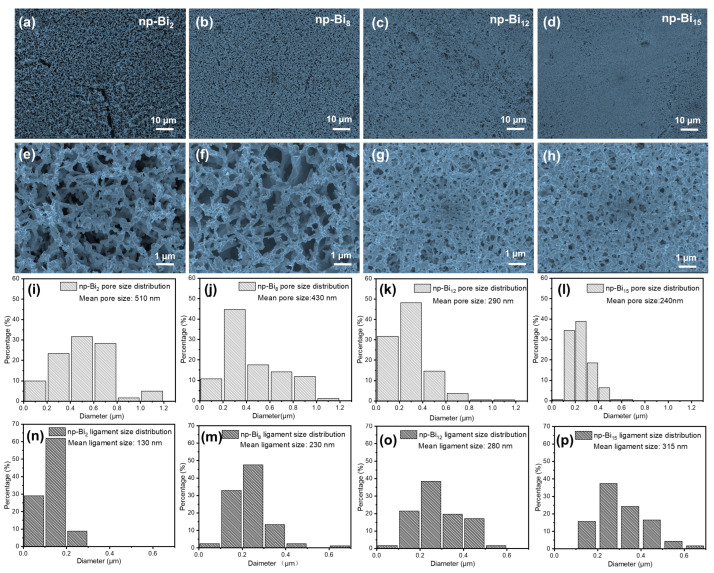
Top-view SEM images of as-dealloyed np-Bi samples form different composition parent alloys, Pb_98_Bi_2_ (**a**,**e**), Pb_88_Bi_12_ (**b**,**f**), Pb_92_Bi_8_ (**c**,**g**), Pb_85_Bi_15_ (**d**,**h**), and its corresponding pore/ligament size distribution map (**i**–**p**). Applied potential is −0.55 V vs. MSE. Electrolyte is 0.5 M HNO_3_ aqueous solution, dealloying temperature is 10 °C.

SEM images were used to investigate the changes in pore/ligament sizes and structural transformations of np-Bi of four selected bismuth content after dealloying at 10 °C. The results show rapid shifts in the millimeter-size geometric porous structure of nanoporous bismuth. The low magnification SEM image (Figure 3a–d) shows that as the composition of the parent alloy increases from 2 to 15 atom%, the average pore diameter of dealloyed Bi gradually decreases, while the pore structure becomes more intact. The np-Bi_2_ sample (Figure 3a) exhibits a few cracks, indicating that it undergoes large structural shrinkage and becomes very fragile when the Bi content is too low. On the other hand, the high magnification SEM image (Figure 3e–h) clearly reveals various pore structures for the four composite parent alloy samples. The above figure shows that all four parent alloy samples with different components exhibit a typical bi-continuous nanoporous structure with relatively rough surface pore walls under the same reaction conditions. As the bismuth content increases, the pore wall structure becomes more connected to form a cohesive whole, while decreasing the Bi content of parent alloy results in a decrease in connectivity and a change in porous structure morphology (Figure 3e). The np-Bi_2_ porous structure has the weakest connectivity and appears empty and foamy, exhibiting a root-like nanowire morphology with a width of approximately 30 nm and a length of 500 nm (Figure S5, Supplementary Materials).

Nano Measure software was used to measure the pore/ligament size of the sample and its distribution. It can be found that the maximum pore size of almost all components of the sample is less than 1200 nm. It is worth noting that only a few cases of pore size greater than 1200 nm could be observed in the np-Bi_8_ sample. The open-door convey structure of np-Bi_8_ has a similar foamy structure to np-Bi_2_ with an average pore size of 430 nm and a ligament size of 230 nm. The pore size distribution tends to be more uniform. Notably, the np-Bi_2_ sample (Figure 3i,n) exhibits a hierarchical pore structure with an average pore size of 510 nm and a very small ligament size of 130 nm. As the Bi atomic content increases to 12 at%, the pore size gradually decreases to a minimum of 290 nm (Figure 3k,o), while ligament size increases from 130 to 280 nm, and the differences in pore and ligament sizes become less pronounced, exhibiting a rougher and curve structure compared to np-Bi_2_ and np-Bi_8_. However, the np-Bi_15_ sample has the narrowest pore size distribution of 240 nm, but the distribution of ligament sizes is wider with a mean size of 315 nm (Figure 3i,p). This suggests an inflection point in the geometric porous structure after dealloying at a bismuth atomic ratio of 12%. At this atomic ratio, the channels and pore walls appear to be most uniform, interconnected with sufficient surface roughness, while still maintaining several large channels (800–1000 nm) (Figure S6, Supplementary Materials) This unique structure has the potential to expose a large number of edge atoms and facilitate quick charge transfer while maintaining excellent structural integrity.

### 3.2. The CO_2_RR Activity of Np-Bi Samples with Different Morphology

**Figure 4:**
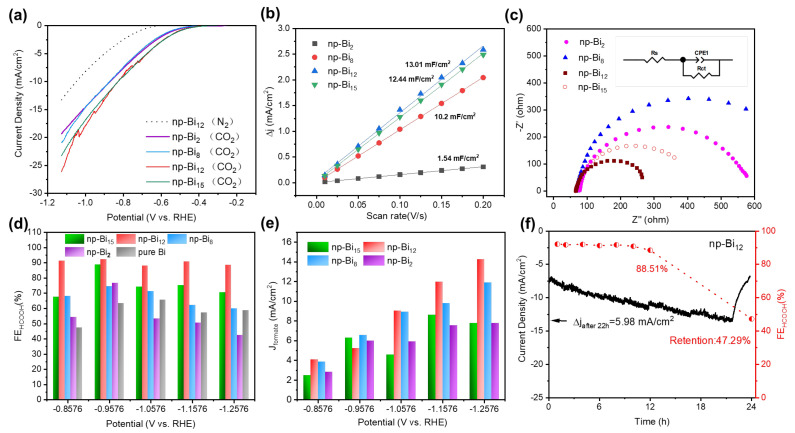
(**a**) The LSV curve of np-Bi electrodes under N_2_ or CO_2_ gas saturation. (**b**) Linear fitting ECSA value of four different nanoporous Bi electrodes, (**c**) Nyquist plots for nanoporous Bi with four different morphologies. (**d**) The performance of the electrocatalytic reduction of carbon dioxide by four nanoporous Bi samples with different morphologies. (**e**) The partial current densities of formic acid. (**f**) Long-term stability test of np-Bi_12_.

The CO_2_RR performances of the prepared nanoporous electrodes were systematically evaluated in CO_2_-saturated 0.1 M KHCO_3_ electrolyte using an H-type cell. LSV analysis was conducted to investigate the influence of different morphologies on the competing hydrogen evolution reaction (HER) (Figure 4a). The onset potentials and cathodic currents for all four samples in CO_2_-saturated electrolyte is significantly more positive than those observed in N_2_-saturated electrolyte. Specifically, the onset potential of np-Bi electrodes in CO_2_-saturated electrolyte is 350 mV more positive than that of samples in N_2_ atmosphere, which has resulted in a dramatic increase in the current density, suggesting that np-Bi electrodes have a higher catalytic activity toward CO_2_RR rather than hydrogen evolution reaction (HER). Furthermore, the np-Bi_12_ and np-Bi_15_ electrodes with a more uniform and curve structure exhibit higher onset potentials and current densities than np-Bi_2_ and np-Bi_8_ electrodes. Among the four samples, np-Bi_12_ shows the highest current density for CO_2_RR, at 26.2 mA cm^−2^ with −1.2 V vs. RHE compared to np-Bi_2_ (19.3mA cm^−2^), np-Bi_8_ (20.8 mA cm^−2^), and np-Bi_15_ (23.2 mA cm^−2^), indicating that the structural uniformity can reveal numerous active sites with high intrinsic catalytic activity. Periodic fluctuations of current density were observed for np-Bi_12_, which may be attributed to the high catalytic activity of np-Bi_12_, leading to the efficient utilization of active sites but relatively poor mass transport. The electrochemically active surface area (ECSA) of four different morphologies of np-Bi were compared by analyzing their double-layer capacitances (C_dl_) obtained from cyclic voltammetry (CV) tests to confirm their electrocatalytic activities (Figure S7, Supplementary Materials). The ECSA generally increase with increasing Bi content, with np-Bi_12_ exhibiting the highest calculated C_dl_ value of 13.01 mF cm^−2^. This is almost a 9-fold increase from the hierarchically structured np-Bi_2_ to the uniform np-Bi_12_. The np-Bi_15_ sample showed a slightly deteriorating C_dl_ value of 12.44 mF cm^−2^; this may be due to variations in the ligament size distribution (Figure 4b). The results suggest that the uniform shape of the nanopores and connection ligaments in np-Bi_12_ not only produces heightened roughness on its edge sites but also facilitates the penetration of the electrolyte, increasing the contact area between the catalyst and electrolyte. This enhances the accessibility of active sites for CO_2_ adsorption, activation, and reduction. Moreover, as displayed in the Nyquist plots (Figure 4c) measured by electrochemical impedance spectroscopy (EIS) analysis, the charge transfer resistance of the four electrodes varies with increased Bi content in parent alloys, the Nyquist plots of np-Bi_8_ and np-Bi_2_ electrodes show large arc sizes compared with np-Bi_12_ and np-Bi_15_ electrodes. The most uniform np-Bi_12_ electrodes exbibit the lowest arcs size out of all the samples, indicating that the high-efficiency charge transfer phenomenon happened in the CO_2_RR process. These outcomes highlight the importance of preserving the integral uniform structure of np-Bi_12_ in maximizing catalytic activity.

Electrolysis tests were conducted to evaluate the different morphologies of np-Bi samples as a catalyst for formic acid generation and uncover the structure–performance correlations. As shown in Figure 4d, the formate faradaic efficiencies (FEs) of four np-Bi electrodes all demonstrate an initial increase followed by a decline at higher potentials, with all catalysts reaching their maximum efficiency at −0.956V. The np-Bi_12_ sample displays the highest selectivity for HCOO^−^ with a faradaic efficiency of 92.16%, while the np-Bi_15_ sample only achieves a selectivity of 88.8%. In contrast, the np-Bi_8_ and np-Bi_2_ samples exhibited lower efficiencies due to structural connectivity loss and larger pore size, resulting in maximum efficiencies of 74.5% and 76.7%, respectively (Table S1, Supplementary Materials). To demonstrate the superior activity of np-Bi, we conducted bulk pure Bi plates of the same size as a blank control group. As shown in Figure 4e, pure Bi plates achieve only 40–60% faradaic efficiency over the entire potential range with the highest FE reaching only 65%, which is much lower than that of the as-synthesized np-Bi_12_ electrodes. This suggests that the nanoporous np-Bi structure exhibits a higher intrinsic catalytic activity and provides abundant active sites, which facilitate CO_2_ absorption and lower the *OCHO binding energy required for the generation of formic acid instead of hydrogen evolution reaction (HER). However, it is important to note that an inappropriate geometric nanoporous structure such as np-Bi_2_ may not make full use of these active sites. Interestingly, the np-Bi_12_ electrode maintains a consistently high faradaic efficiency (>88%) from −0.85 to −1.25V, showing a wide window of highly efficiency potential of 400 mV. In contrast, high selectivity for HCOO^−^ (>80%) in the np-Bi_15_ electrode is only achieved within a narrow potential range of ~100 mV. This phenomenon may be attributed to the highly uniform bi-continuous ligament/pore structures. The unique roughness and highly curved surface of the np-Bi_12_could increase the concentration of CO_2_ near the active sites and enhance the adsorption of the CO_2_ intermediates [17,36]. 

Figure S8 (Supplementary Materials) shows that the total current density of np-Bi electrodes increases as the applied potential becomes more negative. Compared to a pure Bi plate, even with an applied potential shift of −1.256 V vs. RHE, np-Bi electrodes exhibit significantly higher current densities, with the pure Bi plate’s current density never surpassing 9 mA cm^−2^, suggesting that the unique nanoporous structures play a crucial role in enhancing the electrocatalytic performance. During the electrolysis process, minimal fluctuations in current density are observed for the np-Bi_8_ sample, whereas the np-Bi_12_ sample displays significant oscillations in current density, which is consistent with the LSV curves. The np-Bi_2_ and np-Bi_8_ samples exhibit high total current densities above 13 mA cm^−2^ at −1.156 V and −1.256 V, reaching a maximum value of 18.3 and 19.8 mA cm^−2^, respectively. Meanwhile, the total current density of np-Bi_12_ and np-Bi_15_ only achieves the highest values of 16.1 and 10.7 mA cm^−2^. The faradaic efficiency of np-Bi_2_ and np-Bi_8_ for formic acid is very low, resulting in the distribution of a large amount of catalytic current to generate other products. This implies that the nanoporous structures of np-Bi_2_ and np-Bi_8_ electrodes also exhibit certain catalysis activity on the contact surface but are hindered by the competitive reactivity of HER at high potentials, which reduces the selectivity of formic acid. Figure 4e summarizes the partial current densities of formic acid of four np-Bi electrodes, which agrees with our theory, with np-Bi_12_ achieving the highest partial current density of 14.2 mA cm^−2^ at −1.25 V compared to other np-Bi electrodes.

A constant electrolysis test was conducted at −0.965 V vs. RHE for 24 h to assess the long-term performance (Figure 4f) and structural stability of np-Bi_12_ in CO_2_ reduction reaction (CO_2_RR). A CO_2_-saturated 0.1 M KHCO_3_ aqueous solution was used as an electrolyte. The np-Bi_12_ electrode displayed stable activity over the first 12 h of the experiment, with minor fluctuations in faradaic efficiency above 88.5%. The current density showed larger fluctuations due to the accumulation of gaseous products on the surface of electrodes caused by the continuous purging of CO_2_ in the cathodic compartment at a flow rate of 40 mL min^−1^. After 24 h of electrolysis, the faradaic efficiency of formate decreases to 47.29%. To investigate the possible changes in morphology and structure after the long-term stability test, SEM and EDS analyses were performed (Figure S9, Supplementary Materials). The SEM images display various small nanosheets covering the porous surface. The EDS pattern and atomic ratio calculations indicated that Bi_2_O_3_ nanosheets had grown on the nanoporous surface. However, the effects of these nanosheets will differ from those produced in the reference study, where a Bi/Bi_2_O_3_ structure supported formate production [37,38]. This demonstrates the excellent stability of np-Bi_12_ under continuous catalytic conditions over a 12 h period.

## 4. Conclusions

In conclusion, a cost-effective and production-scalable one-step electrochemical dealloying method has been successfully developed to fabricate monolith 3D bi-continuous nanoporous bismuth electrodes for efficient CO_2_ reduction. During selective electrochemical dealloying, lead elements are firstly etched out of the Pb-Bi surface, and the high surface diffusion rate of bismuth atoms at room temperature over the alloy surface leads to the growth of 3D hierarchical nanoporous bismuth with a preferred growing orientation downwards from the dealloying surface. The geometric structure and pore size of the nanoporous bismuth electrode can be readily controlled by varying the dealloying parameters. The fine engineered inter-connected uniform ligament/pore nanoporous bismuth-based electrodes (np-Bi_12_) receive abundant active sites, lower charge transfer resistance and better intrinsic activity compared with other np-Bi electrodes. The np-Bi_12_ catalyst exhibited exceptional electrocatalytic properties, including a high formate faradaic efficiency of 92.6% at −0.956 V vs. RHE, a high current density of 14.2 mA cm^−2^ at −1.25 V vs. RHE, and a wide potential window of 400 mV for selective CO_2_ electrochemical conversion. This innovative method of introducing monolith 3D bi-continuous micro-nanoporous bismuth can lead to insights into the design of advanced 3D nanomaterials for sustainable energy technologies.

## Data Availability

Not applicable.

## Supplementary Materials

The following supporting information can be downloaded at: https://www.mdpi.com/article/10.3390/nano13111767/s1, Figure S1: Linear sweep voltammetry curves of Pb_98_Bi_2_, Pb_92_Bi_8_, Pb_88_Bi_12_, and Pb_85_Bi_15_ alloy; Figure S2: EDS analyses of Pb_98_Bi_15_ alloy before (a) and after dealloying (b) and (c) element qualitative analysis; Figure S3: Cross-section SEM images of np-Bi_12_ sample; Figure S4: Top-view SEM images of Pb_98_Bi_2_ alloy after the potentiostatic dealloying at 10 °C, 45 °C and 80 °C; Figure S5: (a,b) High-resolution scanning electron micrograph of np-Bi_2_ sample; Figure S6: Distribution diagram of the average pore size and ligament size of nanoporous Bi samples as a function of composition; Figure S7: Cyclic voltammetry (CV) curves of np-Bi_2_, np-Bi_8_, np-Bi_12_, and np-Bi_15_ at different scanning speeds; Figure S8: Time-dependent current density curves of np-Bi12 and np-Bi_8_at different potentials and the calculated total current density (J_total_) values of four nanoporous electrodes; Figure S9: Low and high magnification SEM images of np-Bi_12_ electrodes after 24 h of long-term electrolysis test; Figure S10: 1H NMR spectrum for qualitative determination of formate product as well as the related calibration curve; Table S1: The relationship between the size of four nanoporous Bi samples with different morphologies and the faradaic efficiency of formic acid.
