# Supplementary material for: One-Step Electrochemical Dealloying of 3D Bi-Continuous Micro-Nanoporous Bismuth Electrodes and CO2RR Performance
Source: Nanomaterials (Basel). 2023 May 30;13(11):1767. doi: 10.3390/nano13111767 (PMC10254552; doi:10.3390/nano13111767)
Supplement: Supplementary file 1 [file nanomaterials-13-01767-s001.zip › nanomaterials-2422026-supplementary.pdf]

Supporting Information for:

One-Step Electrochemical Dealloying of 3D Bi-Continuous Micro-Nanoporous Bismuth Electrodes and CO_2RR Performance

Wenqin Lai [1], Yating Liu [1], Mingming Zeng [1], Dongmei Han [1,2], Min Xiao [1], Shuanjin Wang [1], Shan Ren [1,*] and Yuezhong Meng [1,2,*]

1 The Key Laboratory of Low-Carbon Chemistry & Energy Conservation of Guangdong Province, State Key Laboratory of Optoelectronic Materials and Technologies, School of Materials Science and Engineering, Sun Yat-sen University, Guangzhou 510275, China; laiwq3@mail2.sysu.edu.cn (W.L.); liuyt79@mail2.sysu.edu.cn (Y.L.); zengmm5@mail2.sysu.edu.cn (M.Z.); handongm@mail.sysu.edu.cn (D.H.); stsxm@mail.sysu.edu.cn (M.X.); wangshj@mail.sysu.edu.cn (S.W.)

2 School of Chemical Engineering and Technology, Sun Yat-sen University, Zhuhai 519000, China

* Correspondence: stsrs@mail.sysu.edu.cn (S.R.); mengyzh@mail.sysu.edu.cn (Y.M.)

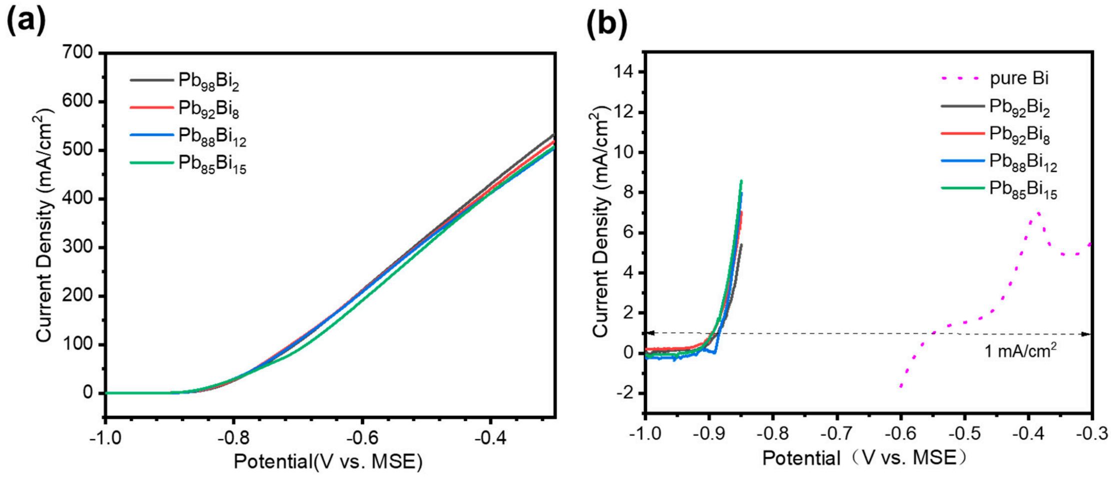

Figure S1 (a) Linear sweep voltammetry curves of $Pb_{98}Bi_2$、$Pb_{92}Bi_8$、$Pb_{88}Bi_{12}$、$Pb_{85}Bi_{15}$ alloy in 0.5 M HNO_3 aqueous solution at 25°C. The scan rate is 5.0 mV/s. (b) Comparison of details of Pb-Bi alloy and pure Bismuth sample in LSV curves.

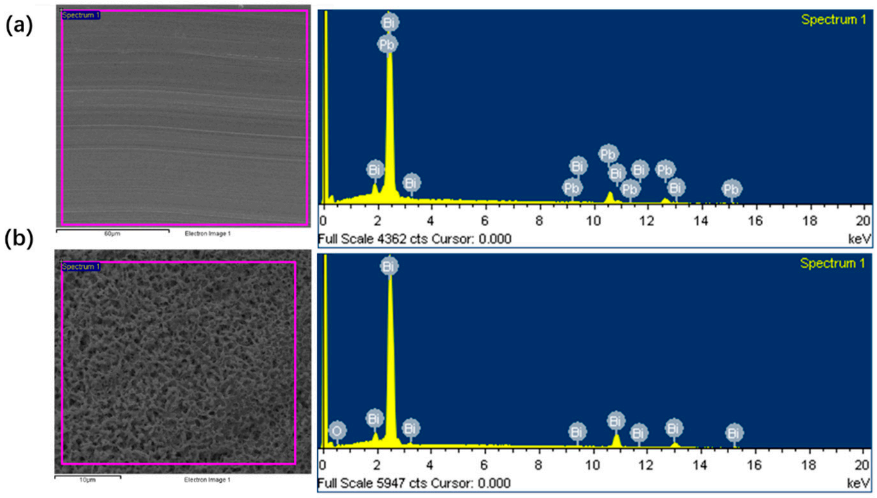

(c)

Element	Weight (%)	Atomic (%)
Pb M	84.10	84.21
Bi M	15.90	15.79
Totals	100%	100%

Figure S2 EDS analyses of $Pb_{98}Bi_{15}$ alloy before (a) and after (b) and (c)element qualitative analysis. the potentiostatic dealloying with electric potential of -0.55 V vs. MSE in 0.5 M HNO_3 aqueous solution at 10℃, 3600s.

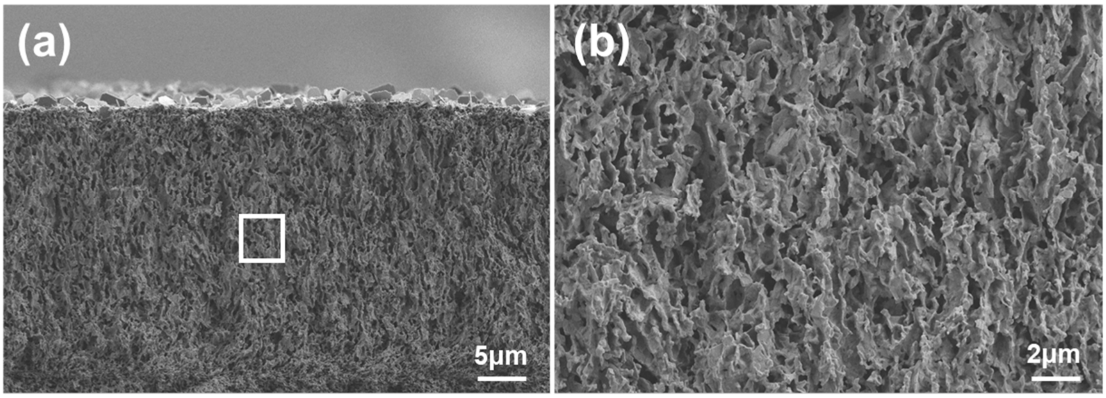

Figure S3 (a,b) cross-section SEM images of np-Bi_{12} sample. dealloying with electric potential of -0.55 V vs. MSE in 0.5 M HNO_3 aqueous solution at 10℃,1200s.

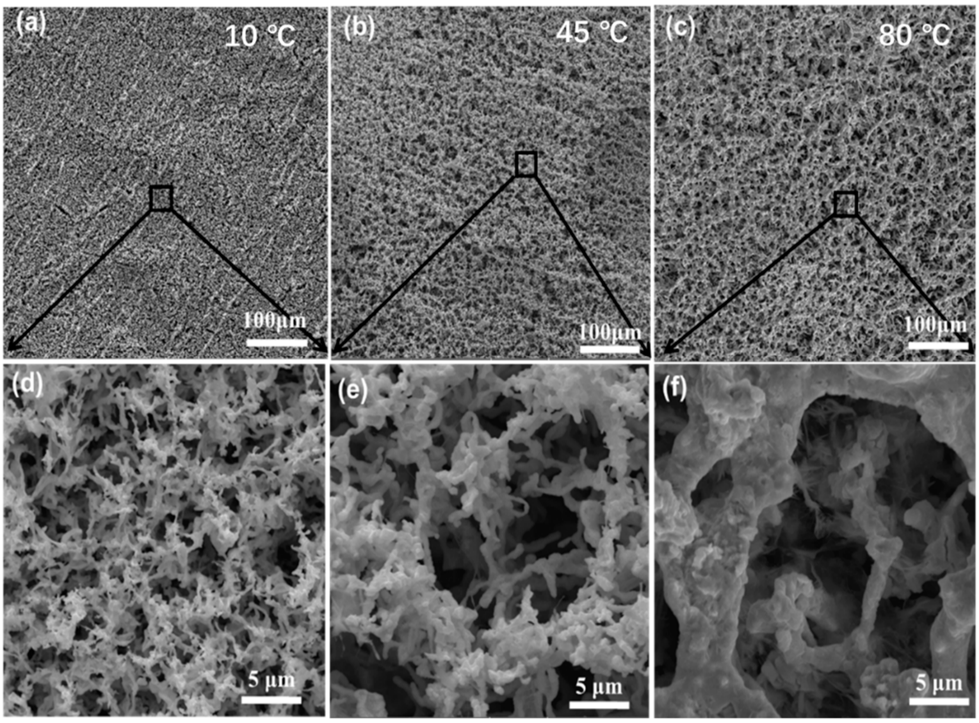

Figure S4 Top-view SEM images of $Pb_{98}Bi_2$ alloy after the potentiostatic dealloying at 10℃ (a,d)、45℃ (b,e) and 80℃ (c,f).

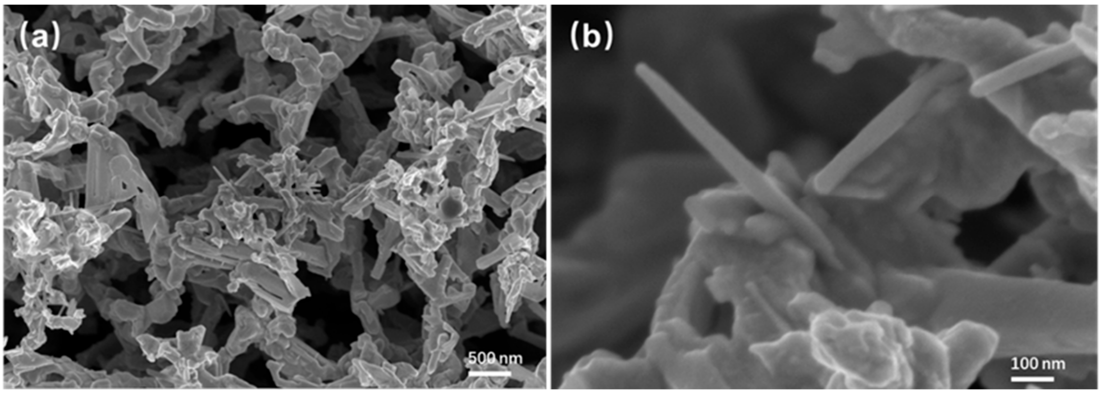

Figure S5 (a,b) High-resolution scanning electron micrograph of np-Bi_2 sample.

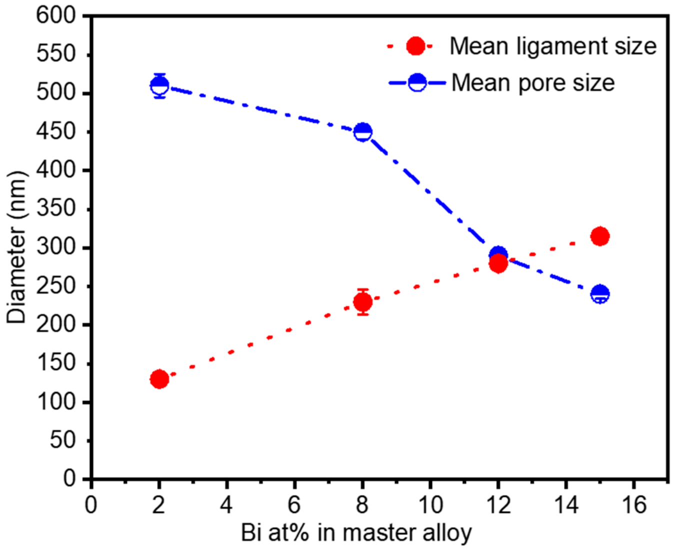

Figure S6 Distribution diagram of the average pore size and ligament size of nano-porous Bi samples as a function of composition. Applied potential is -0.55 V vs. MSE. Electrolyte is 0.5 M HNO_3 aqueous solution, dealloying temperature is 10℃.

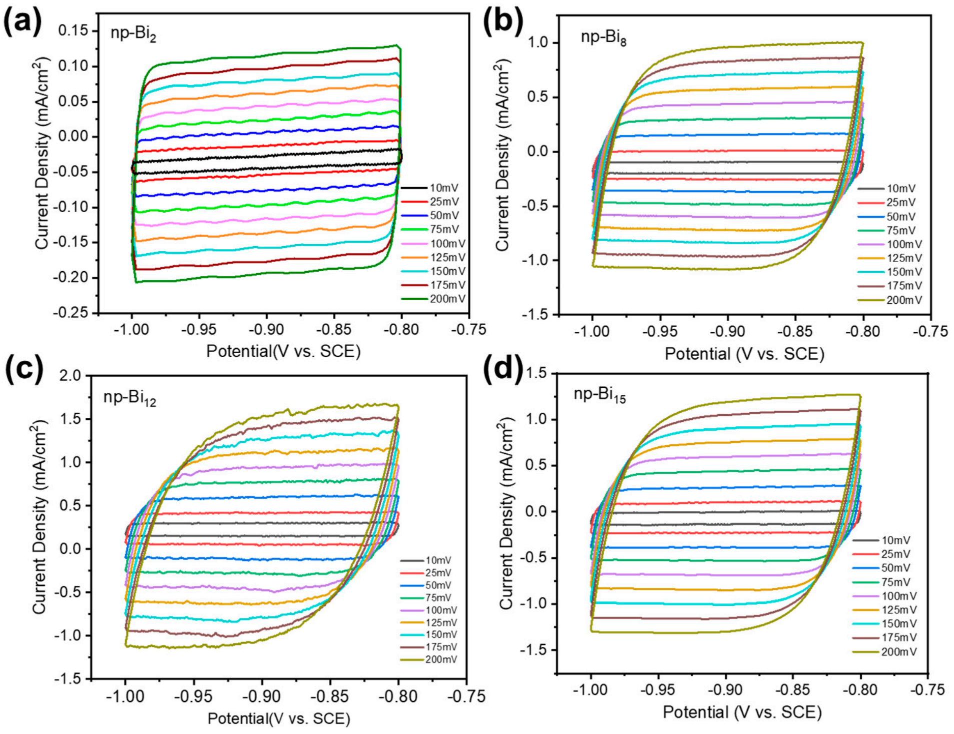

Figure S7 Cyclic voltammetry (CV) curves of （a）np-Bi_2，（b）np-Bi_8，（c）np-Bi_{12}，（d）np-Bi_{15} at different scanning speeds in a 0.1 M $KHCO_3$ aqueous solution saturated with N_2.

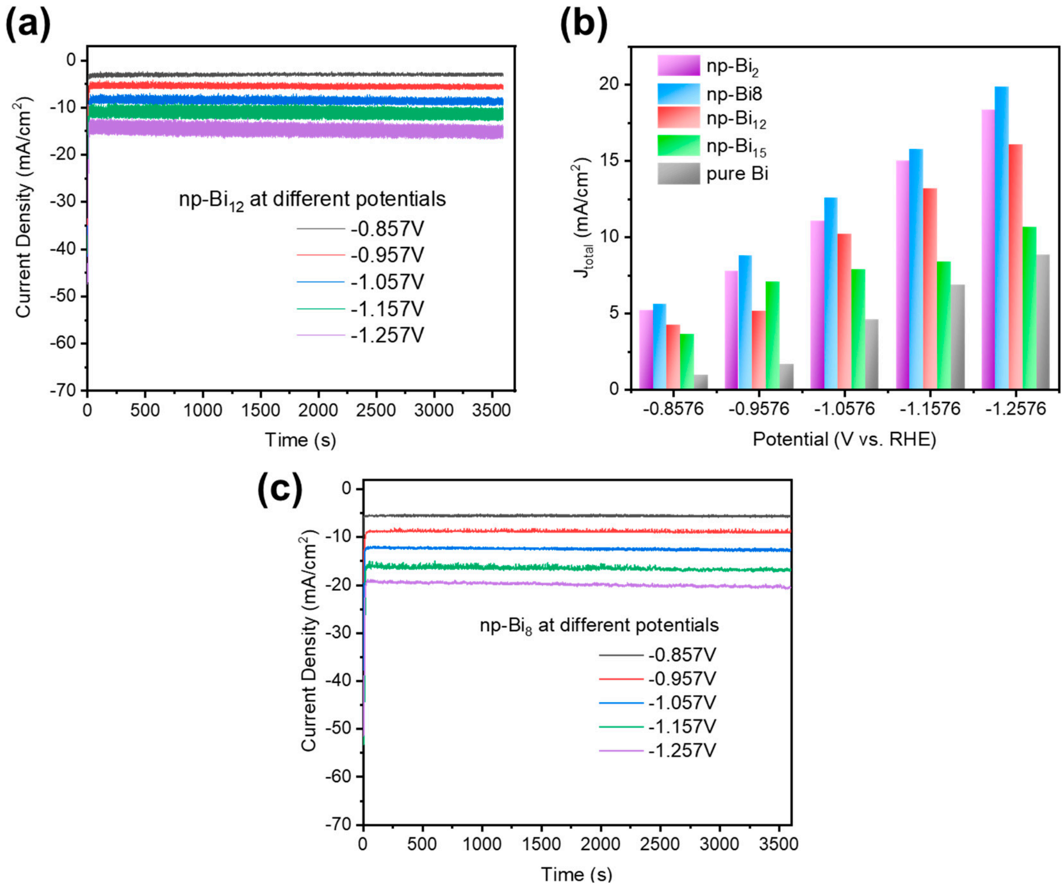

Figure S8 (a) Time-dependent current density curves of np-Bi_{12} at different potentials. (b) The calculated total current density (J_{total}) values of four nanoporous electrodes under 1hour electrochemical catalysis. (c) total current density of np-Bi_8 electrode at different potentials.

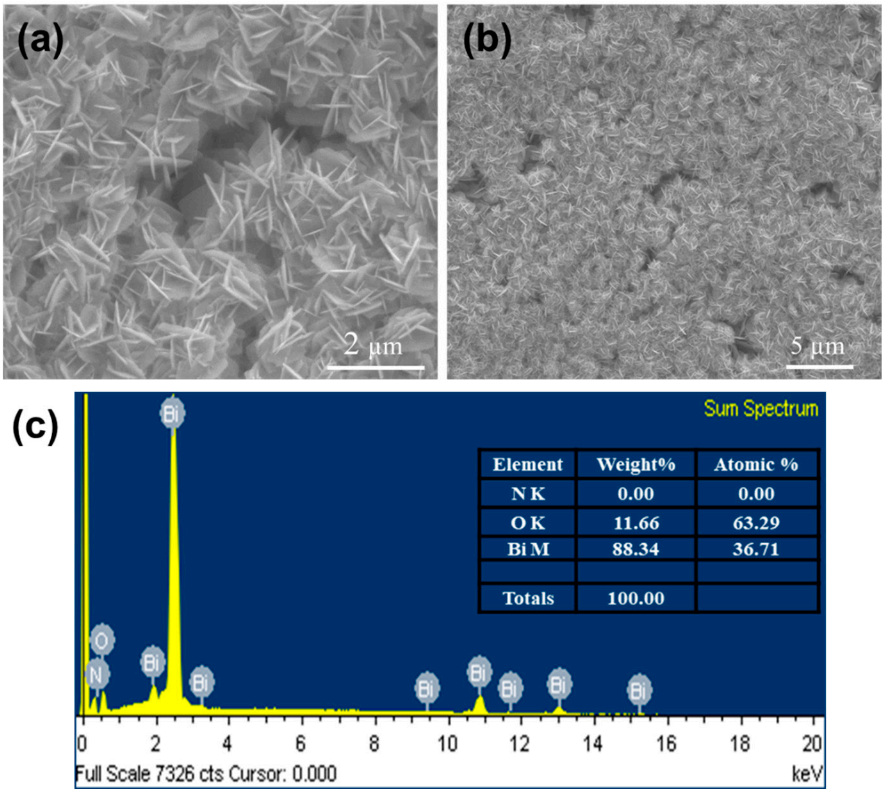

Figure S9 Low (a) and high (b) magnification SEM images of np-Bi_{12} electrodes after 24h of long-term electrolysis test. (c) EDS analysis of the oxidized np-Bi_{12} electrodes.

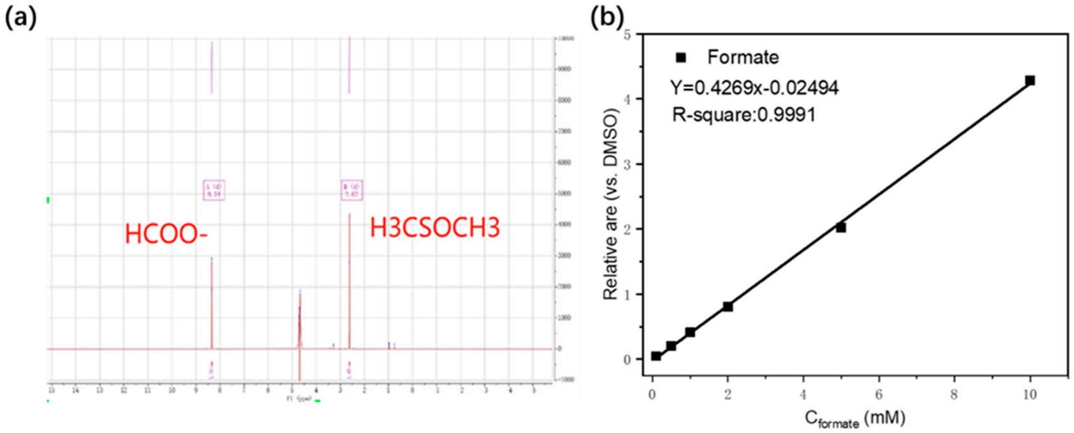

Figure S10 (a) 1H-NMR spectrum for qualitative determination of formate product. (b) Linear relationship between the known concentration of formate and relative area (vs. DMSO). The standard curve was measured from the reference samples prepared by mixing 0.5 mL of HCOONa aqueous solution (0.1, 0.5, 1.0, 2.0, 5.0 and 10 mM concentration) with 0.1 mL D_2O and 0.2 mL 5mM DMSO (an internal standard).

Table S1 The relationship between the size of four nanoporous Bi samples with different morphologies and the Faraday efficiency of formic acid.

Sample Name	Mean pore size （nm）	Mean ligament size（nm）	FE_{HCOOH}（%）
**Bi_2-10**	**510**	**130**	**76.7**
**Bi8-10**	**450**	**230**	**74.5**
**Bi_{12}-10**	**290**	**280**	**92.2**
**Bi_{15}-10**	**240**	**315**	**88.8**
